# Comparative accuracy of spectral computed tomography and conventional computed tomography in colorectal cancer staging

**DOI:** 10.3389/fonc.2026.1802950

**Published:** 2026-06-11

**Authors:** Dan Wei, Jing-Yi Zhang, Ping-Tai Tang, Jin-Kai Liu

**Affiliations:** 1 Department of Radiology, Longyan First Affiliated Hospital of Fujian Medical University, Longyan, Fujian, China; 2 Department of Radiology, Hui Ya Hospital of The First Affiliated Hospital, Sun Yat-sen University, Huizhou, Guangdong, China

**Keywords:** colorectal cancer, diagnostic accuracy, dual-energy computed tomography, spectral computed tomography, tumor–node–metastasis staging

## Abstract

**Background:**

Accurate pre-treatment staging is essential for risk stratification and treatment planning in colorectal cancer (CRC). Spectral computed tomography (CT) provides energy-resolved reconstructions that may improve local and nodal assessment compared with conventional contrast-enhanced CT.

**Methods:**

This retrospective study included 340 adults with pathologically confirmed CRC who underwent spectral CT (n = 151) or conventional CT (n = 189) before definitive treatment between September 2023 and September 2025. The reference standard was postoperative histopathologic staging for resected cases or a composite standard incorporating biopsy, imaging, and clinical follow-up for non-surgical cases. Primary outcomes were overall TNM accuracy and component T, N, and M staging accuracy. Agreement was assessed using kappa statistics. Binary diagnostic performance was calculated for nodal metastasis and distant metastasis. Multivariable logistic regression was performed to account for potential residual confounding.

**Results:**

Spectral CT showed higher overall TNM accuracy than conventional CT (74.2% vs 63.0%, P = 0.028). It also demonstrated superior T-stage accuracy (74.2% vs 57.7%, P = 0.002) and N-stage accuracy (74.8% vs 64.0%, P = 0.032), whereas M-stage accuracy was similar (90.7% vs 91.0%, P = 0.930). Agreement with the reference standard was higher for spectral CT for overall TNM (weighted κ = 0.815 vs 0.795), T stage (0.759 vs 0.663), and N stage (0.599 vs 0.430). For M1 detection, spectral CT showed higher sensitivity (83.8% vs 72.2%) with slightly lower specificity (93.0% vs 95.4%). In adjusted analyses, spectral CT remained independently associated with correct overall TNM staging (adjusted odds ratio 1.91, 95% confidence interval 1.14–3.19, P = 0.014).

**Conclusions:**

Under standardized protocols, spectral CT demonstrated higher overall TNM staging accuracy than conventional CT, primarily through improved T and N classification, while M staging performance was comparable.

## 1 Introduction

Accurate pre-treatment staging is central to colorectal cancer (CRC) management because therapeutic selection depends on the extent of mural invasion, regional nodal involvement, and distant metastatic spread (1, 2). Contemporary clinical pathways typically rely on cross-sectional imaging to define disease extent, stratify prognosis, and guide the sequencing of surgery, systemic therapy, and, where applicable, organ-preserving strategies (3). Practice guidelines continue to emphasize computed tomography (CT) as a cornerstone modality for staging and metastatic surveillance in CRC, while recommending complementary imaging in selected scenarios and for problem solving (4). Conventional contrast-enhanced multidetector CT provides wide anatomic coverage and is broadly accessible, yet its performance for local and nodal staging is constrained by intrinsic limitations (5). Differentiating intermediate T categories can be challenging when tumor margins are indistinct, pericolonic stranding is nonspecific, or adjacent organ interfaces are complex (6). Similarly, CT-based nodal assessment remains imperfect because size and morphology criteria overlap between reactive and metastatic lymph nodes, and small-volume nodal disease may not be detected (7). These limitations have motivated the evaluation of advanced CT techniques that improve contrast resolution and enable quantitative characterization.

Spectral CT (including dual-layer spectral detector CT and other dual-energy implementations) acquires energy-resolved data that can be reconstructed into virtual monoenergetic images (VMIs) and iodine-based material decomposition maps (8, 9). Low-keV VMIs increase iodine conspicuity and may enhance lesion-to-background contrast, potentially improving delineation of the primary tumor and peritumoral changes (10, 11). In parallel, iodine maps and derived quantitative parameters provide surrogate measures of enhancement that may support tissue characterization and reduce interpretive uncertainty, particularly for indeterminate lesions and borderline nodal findings (12, 13). Recent reports have also extended spectral CT applications to clinically relevant metastatic patterns in CRC, including peritoneal metastases detection/quantification and standardized imaging approaches for peritoneal spread, where improved conspicuity could influence staging and treatment planning (14–16).

Despite this rationale, the incremental value of spectral CT over conventional CT for comprehensive CRC staging under standardized institutional protocols remains incompletely defined across T, N, and M components, and the reproducibility of spectral quantitative metrics warrants explicit evaluation. Furthermore, performance may vary by tumor location and disease burden, underscoring the importance of subgroup and sensitivity analyses. The present study therefore investigates CRC staging using spectral CT and conventional CT performed within a predefined pre-treatment interval, with reference standard verification and structured assessment of diagnostic accuracy, agreement, and interobserver reliability.

## 2 Methods

### 2.1 Study design

This retrospective observational study consecutively enrolled patients with pathologically confirmed CRC treated at our institution between September 2023 and September 2025 to compare the staging accuracy of spectral CT and conventional CT. Eligible patients were adults (≥18 years) with a first diagnosis of CRC who had undergone spectral CT or conventional CT performed according to standardized institutional protocols within a predefined interval prior to definitive treatment (surgery and/or neoadjuvant therapy). A verifiable reference standard for staging was required, including postoperative histopathology for resected cases and/or a multidisciplinary consensus incorporating biopsy results and follow-up imaging/clinical data for non-surgical cases. Patients were excluded if they had received any antitumor treatment before CT acquisition (chemotherapy, radiotherapy, or local tumor resection), had synchronous malignancies that could confound staging, had incomplete or non-diagnostic imaging data (incomplete scan coverage, severe motion artifact, or missing contrast-enhanced phases), lacked an appropriate reference standard, or had missing key clinicopathological information necessary for staging comparison. This study was approved by the institutional ethics committee. Informed consent was obtained from all participants. All procedures adhered to relevant guidelines and regulations, in line with the Declaration of Helsinki and its amendments. Participant confidentiality was strictly maintained, with all personal identifiers removed prior to analysis.

### 2.2 CT acquisition

Spectral CT and conventional contrast-enhanced CT examinations were performed according to standardized institutional protocols using a multidetector CT system with spectral capability (IQon Spectral CT, Philips Healthcare). All patients were scanned in the supine position with craniocaudal coverage from the diaphragmatic (or hepatic) dome to the pubic symphysis to ensure complete abdominopelvic evaluation for staging. Acquisition was performed in helical mode with detector collimation of 64 × 0.625 mm, a tube voltage of 120 kV, and automatic tube current modulation (78–145 mAs). The gantry rotation speed was 0.75 s/rotation with a helical pitch of 0.988. Images were reconstructed using a standard soft-tissue kernel with a reconstructed slice thickness of 1.0 mm and a reconstruction interval of 0.5 mm, and multiplanar reconstructions (axial, coronal, and sagittal) were routinely generated. A nonionic iodinated contrast agent (350 mg I/mL) was administered via an antecubital vein using a power injector at 3.0–4.0 mL/s at approximately 1.5 mL/kg (maximum 120 mL), followed by a 30–40 mL saline flush. Portal venous phase imaging (70–80 s after contrast initiation) was mandatory for staging, whereas arterial phase (30–35 s) and delayed phase (~180 s) acquisitions were performed when clinically indicated. For spectral CT, VMIs were reconstructed across 40–140 keV; 70-keV images were used as the default series approximating conventional 120 kVp images, and low-keV images (40–60 keV) were additionally reviewed to improve lesion conspicuity when required.

### 2.3 Image interpretation, TNM definitions, and reference standard

All CT datasets were independently reviewed in an anonymized manner by at least two board-certified radiologists with subspecialty experience in abdominal/gastrointestinal imaging. The two primary readers interpreted all examinations independently, and readers were blinded to the reference standard (pathologic staging and follow-up outcomes) and to each other’s interpretations. All image datasets were anonymized and non-essential clinical information was withheld to minimize expectation and verification bias. Discrepancies between the two primary readers were resolved by consensus; if disagreement persisted, a third senior radiologist served as the adjudicator of the final staging result.

Preoperative staging was performed according to the American Joint Committee on Cancer/Union for International Cancer Control (AJCC/UICC) TNM classification (8th edition). For T staging, the depth of bowel wall invasion was assessed based on CT signs of mural thickening and enhancement, interruption of the normal layered appearance, extension into pericolorectal fat (stranding or nodular infiltration), serosal involvement, and direct invasion of adjacent organs/structures. For N staging, regional lymph nodes were considered suspicious primarily on predefined morphological criteria that were consistently applied by both readers, including a short-axis diameter threshold (typically ≥10 mm) and ancillary features such as a rounded configuration, irregular margins, central low attenuation suggestive of necrosis, and clustering of multiple nodes along the expected drainage pathways. For M staging, distant metastasis was evaluated by the presence of lesions consistent with metastatic disease in the liver, lungs, peritoneum, and nonregional lymph nodes; when available, additional imaging (MRI and/or PET-CT) was considered supportive evidence for confirmation of equivocal distant lesions.

The reference standard was defined *a priori*. For patients undergoing curative-intent resection, postoperative histopathologic TNM staging served as the primary gold standard. For patients without surgical resection, a composite reference standard was applied, incorporating histologic confirmation from biopsy and longitudinal clinical/imaging follow-up to establish metastatic status and overall stage. Pathologic assessment was performed by dedicated gastrointestinal pathologists following institutional procedures; key histopathologic variables, including depth of invasion, number of metastatic lymph nodes, and the presence of lymph vascular or perineural invasion when reported, were recorded to support stage verification.

### 2.4 Outcome measures

Primary outcomes were the accuracies of imaging-based staging. Overall TNM stage accuracy was defined as the proportion of patients whose imaging-assigned overall TNM stage exactly matched the reference standard stage. Component-level accuracy was evaluated separately for T stage, N stage, and M stage, defined as exact agreement between imaging-based category (T, N, or M) and the corresponding reference standard category. In addition to accuracy, stage agreement was assessed to characterize concordance between imaging and the reference standard at both the overall-stage level and the individual T, N, and M components.

Secondary outcomes included standard diagnostic performance metrics in clinically meaningful binary classification settings. Specifically, sensitivity, specificity, positive predictive value (PPV), negative predictive value (NPV), and overall accuracy were calculated for: (1) locally advanced primary tumor (e.g., T ≥ 3 vs. T1–2), (2) regional nodal involvement (N+ vs. N0), and (3) distant metastasis (M1 vs. M0). These binary endpoints were selected to reflect key decision points in preoperative management (selection of neoadjuvant therapy and determination of surgical strategy).

Agreement analyses were performed to evaluate reliability. For categorical staging variables, agreement was quantified using Cohen’s kappa with 95% confidence intervals. For ordinal outcomes (e.g., T stage or overall stage), weighted kappa was applied to account for the degree of disagreement across ordered categories. If quantitative spectral parameters were included (e.g., iodine concentration or normalized iodine indices), interobserver reproducibility was assessed using the intraclass correlation coefficient (ICC) with 95% confidence intervals based on an appropriate two-way model.

### 2.5 Statistical analysis

All statistical analyses were performed using IBM SPSS Statistics, version 26.0 (IBM Corp., Armonk, NY, USA). Continuous variables were assessed for distributional characteristics and are presented as mean ± standard deviation when approximately normally distributed; between-group comparisons were conducted using the independent-samples t test, and Welch’s t test was applied when variance heterogeneity was identified. Categorical variables are presented as number (percentage) and were compared using the Pearson chi-square test; Fisher’s exact test was used when expected cell counts were small. Staging performance was evaluated against the prespecified reference standard. Accuracy was defined as the proportion of cases with exact agreement between imaging-based staging and the reference standard for overall TNM stage and for T, N, and M components. For clinically relevant binary endpoints, including locally advanced disease (T ≥ 3 vs T1–2), nodal metastasis (N+ vs N0), and distant metastasis (M1 vs M0), diagnostic performance indices were calculated, including sensitivity, specificity, positive predictive value, negative predictive value, and accuracy. Agreement analyses were performed to assess reliability. Interobserver agreement for categorical staging outcomes was quantified using Cohen’s kappa, and weighted kappa was used for ordinal outcomes. For continuous spectral parameters, interobserver reproducibility was assessed using the intraclass correlation coefficient. Multivariable logistic regression analyses were performed with correct overall TNM staging (1 = correct, 0 = incorrect) as the dependent variable and imaging modality as the primary independent variable. Model 1 adjusted for age, sex, tumor size, tumor location, histologic differentiation, stage grouping, and treatment intent; Model 2 additionally adjusted for reference standard type. Adjusted odds ratios (aORs) with 95% confidence intervals (CIs) were reported, and multicollinearity was assessed using variance inflation factors. All statistical tests were two-sided, and a P value < 0.05 was considered statistically significant.

## 3 Results

### 3.1 Patient enrollment

A total of 353 patients with pathologically confirmed CRC were initially identified from the institutional pathology database and the picture archiving and communication system (PACS). After applying the prespecified eligibility criteria, 13 patients were excluded for the following reasons: incomplete or non-diagnostic imaging data (n = 5), including incomplete scan coverage, missing essential contrast-enhanced phases, or severe motion artifacts precluding reliable staging; CT examination performed outside the predefined pre-treatment interval (n = 3); absence of an appropriate reference standard (n = 3), defined as lack of postoperative pathological TNM staging and insufficient follow-up to establish a composite reference standard; and prior anticancer treatment, recurrent disease, or synchronous malignancy that could confound staging assessment (n = 2). Ultimately, 340 patients were included in the final analysis and were categorized according to the preoperative imaging modality used for staging: the spectral CT group (n = 151) and the conventional CT group (n = 189).

### 3.2 Baseline characteristics and tumor profile

Patients in the spectral CT group had a mean age of 62.77 ± 9.78 years, compared with 64.44 ± 9.82 years in the conventional CT group (t = −1.55, P = 0.122). The proportion of male patients was 61.6% (93/151) in the spectral CT group and 58.2% (110/189) in the conventional CT group (χ² = 0.40, P = 0.527). Mean BMI was identical between groups (24.10 ± 3.06 vs. 24.10 ± 3.23 kg/m²; t = 0.02, P = 0.987). The mean tumor size was 4.09 ± 1.59 cm in the spectral CT group and 4.31 ± 1.68 cm in the conventional CT group (t = −1.24, P = 0.214). Tumor location distributions (right colon/left colon/rectum) did not differ significantly between groups (χ² = 2.30, P = 0.316), nor did histologic differentiation (well/moderate/poor) (χ² = 0.83, P = 0.662). The rate of laparoscopic surgery was 68.9% (104/151) in the spectral CT group and 71.4% (135/189) in the conventional CT group (χ² = 0.26, P = 0.609). Overall, no statistically significant between-group differences were observed across baseline demographic or tumor-related variables (all P > 0.05), indicating acceptable comparability between groups (**Table 1**).

**Table 1:** Baseline demographic and clinicopathologic characteristics.

Variable	Spectral CT (n = 151)	Conventional CT (n = 189)	Test statistic	P value
Age, years	62.77 ± 9.78	64.44 ± 9.82	t = −1.55	0.122
Male sex, n (%)	93 (61.6)	110 (58.2)	χ² = 0.40	0.527
BMI, kg/m²	24.10 ± 3.06	24.10 ± 3.23	t = 0.02	0.987
Tumor size, cm	4.09 ± 1.59	4.31 ± 1.68	t = −1.24	0.214
Tumor location (Right/Left/Rectum), n (%)	65 (43.0)/52 (34.4)/34 (22.5)	71 (37.6)/62 (32.8)/56 (29.6)	χ² = 2.30	0.316
Differentiation (Well/Moderate/Poor), n (%)	33 (21.9)/86 (57.0)/32 (21.2)	36 (19.0)/106 (56.1)/47 (24.9)	χ² = 0.83	0.662
Laparoscopic surgery, n (%)	104 (68.9)	135 (71.4)	χ² = 0.26	0.609

BMI, body mass index; CT, computed tomography; SD, standard deviation.

### 3.3 Overall TNM staging accuracy and agreement with the reference standard

Overall TNM staging accuracy was 74.2% (112/151) in the spectral CT group and 63.0% (119/189) in the conventional CT group, indicating a higher proportion of exact TNM stage matches with the reference standard in the spectral CT cohort (χ² = 4.84, P = 0.028). Agreement with the reference standard was high for both modalities, with a quadratic weighted κ of 0.815 (95% CI 0.742–0.874) for spectral CT and 0.795 (95% CI 0.741–0.838) for conventional CT (**Table 2**).

**Table 2:** Comparison of overall TNM staging performance.

Outcome	Spectral CT (n = 151)	Conventional CT (n = 189)	Test statistic	P value
Overall TNM stage accuracy, n (%)	112 (74.2)	119 (63.0)	χ² = 4.84	0.028
Quadratic weighted κ (95% CI)	0.815 (0.742–0.874)	0.795 (0.741–0.838)	—	—

CI, confidence interval; CT, computed tomography; κ, kappa.

### 3.4 Multivariable logistic regression analysis for correct overall TNM staging

To further account for potential residual confounding related to the non-random assignment of imaging modality, we performed two multivariable logistic regression models with correct overall TNM staging as the dependent variable (**Supplementary Table 1**). In Model 1, after adjustment for age, sex, tumor size, tumor location, histologic differentiation, reference-standard stage grouping, and treatment intent, spectral CT remained independently associated with higher odds of correct overall TNM staging compared with conventional CT (aOR 1.91, 95% CI 1.14–3.19; P = 0.014). Advanced stage (III–IV vs I–II) was associated with lower odds of correct staging (aOR 0.58, 95% CI 0.34–0.98; P = 0.042), whereas age, sex, tumor size, tumor location, histologic differentiation, and treatment intent were not independently associated with staging correctness. In Model 2, after additional adjustment for reference standard type, the association between spectral CT and correct overall TNM staging remained materially unchanged (aOR 1.87, 95% CI 1.10–3.17; P = 0.019). Reference standard type itself was not significantly associated with correct staging (aOR 0.76, 95% CI 0.40–1.44; P = 0.398). The consistency of the estimates across the two models supported the robustness of the main finding.

### 3.5 T, N, and M staging performance

Spectral CT demonstrated higher concordance with the reference standard than conventional CT for both T and N staging (**Table 3**). For T stage, accuracy was 74.2% (112/151) in the spectral CT group versus 57.7% (109/189) in the conventional CT group (χ² = 10.05, P = 0.002). Agreement was also higher with spectral CT (quadratic weighted κ = 0.759, 95% CI 0.666–0.844) than with conventional CT (κ = 0.663, 95% CI 0.567–0.746). For N stage, accuracy was 74.8% (113/151) versus 64.0% (121/189), respectively (χ² = 4.57, P = 0.032), with higher agreement for spectral CT (κ = 0.599, 95% CI 0.490–0.702) compared with conventional CT (κ = 0.430, 95% CI 0.325–0.531). In contrast, M-stage accuracy was comparable between groups (90.7% [137/151] vs 91.0% [172/189]; χ² = 0.01, P = 0.930), with similar agreement (spectral CT κ = 0.754, 95% CI 0.619–0.869; conventional CT κ = 0.699, 95% CI 0.552–0.821).

**Table 3:** T-, N-, and M-stage accuracy and agreement with the reference standard.

Stage component	Spectral CT (n = 151)	Conventional CT (n = 189)	Test statistic	P value
T stage accuracy, n (%)	112 (74.2)	109 (57.7)	χ² = 10.05	0.002
T stage agreement, quadratic weighted κ (95% CI)	0.759 (0.666–0.844)	0.663 (0.567–0.746)	—	—
N stage accuracy, n (%)	113 (74.8)	121 (64.0)	χ² = 4.57	0.032
N stage agreement, Cohen’s κ (95% CI)	0.599 (0.490–0.702)	0.430 (0.325–0.531)	—	—
M stage accuracy, n (%)	137 (90.7)	172 (91.0)	χ² = 0.01	0.930
M stage agreement, Cohen’s κ (95% CI)	0.754 (0.619–0.869)	0.699 (0.552–0.821)	—	—

CI, confidence interval; CT, computed tomography; κ, kappa.

Binary analyses showed similar sensitivity for N+ detection (85.9% vs 88.2%) but higher specificity with spectral CT (73.8% vs 67.3%). For M1 detection, spectral CT had higher sensitivity (83.8% vs 72.2%) with slightly lower specificity (93.0% vs 95.4%) (**Table 4**).

**Table 4:** Binary diagnostic performance for N+ and M1.

Endpoint and metric	Spectral CT, % (95% CI)	Conventional CT, % (95% CI)
Nodal metastasis (N+ vs N0)		
Sensitivity	85.9 (76.0–92.2)	88.2 (79.7–93.5)
Specificity	73.8 (63.2–82.1)	67.3 (57.8–75.6)
PPV	74.4 (64.0–82.6)	68.8 (59.6–76.7)
NPV	85.5 (75.3–91.9)	87.5 (78.5–93.1)
Accuracy	79.5 (72.3–85.1)	76.7 (70.2–82.2)
Distant metastasis (M1 vs M0)		
Sensitivity	83.8 (68.9–92.3)	72.2 (56.0–84.2)
Specificity	93.0 (86.8–96.4)	95.4 (90.9–97.8)
PPV	79.5 (64.5–89.2)	78.8 (62.2–89.3)
NPV	94.6 (88.8–97.5)	93.6 (88.6–96.5)
Accuracy	90.7 (85.0–94.4)	91.0 (86.1–94.3)

CI, confidence interval; CT, computed tomography; NPV, negative predictive value; PPV, positive predictive value.

### 3.6 Interobserver agreement and reader reliability

Interobserver agreement between the two readers was assessed separately for each staging component. For overall TNM stage, quadratic weighted κ was 0.779 (95% CI 0.702–0.842) in the spectral CT group and 0.760 (95% CI 0.668–0.825) in the conventional CT group (both P < 0.001). For T stage, agreement was 0.819 (95% CI 0.757–0.872) and 0.763 (95% CI 0.684–0.825), respectively (both P < 0.001). Agreement for N stage was κ = 0.600 (95% CI 0.487–0.708) for spectral CT and κ = 0.530 (95% CI 0.427–0.624) for conventional CT (both P < 0.001). For M stage, κ was 0.643 (95% CI 0.498–0.771) and 0.615 (95% CI 0.447–0.751), respectively (both P < 0.001). For the continuous spectral parameter, interobserver reproducibility of iodine concentration in the spectral CT group was ICC = 0.943 (95% CI 0.921–0.958; P < 0.001) (**Table 5**).

**Table 5:** Interobserver agreement for staging and reproducibility of a continuous spectral parameter.

Measure	Spectral CT (n = 151)	Conventional CT (n = 189)	P value
Overall TNM stage, quadratic weighted κ (95% CI)	0.779 (0.702–0.842)	0.760 (0.668–0.825)	<0.001
T stage, quadratic weighted κ (95% CI)	0.819 (0.757–0.872)	0.763 (0.684–0.825)	<0.001
N stage, Cohen’s κ (95% CI)	0.600 (0.487–0.708)	0.530 (0.427–0.624)	<0.001
M stage, Cohen’s κ (95% CI)	0.643 (0.498–0.771)	0.615 (0.447–0.751)	<0.001
Iodine concentration (spectral parameter), ICC (95% CI)	0.943 (0.921–0.958)	—	<0.001

CI, confidence interval; CT, computed tomography; ICC, intraclass correlation coefficient; κ, kappa; TNM, tumor–node–metastasis.

### 3.7 Subgroup analyses of overall TNM staging accuracy

In location-stratified analyses, overall TNM accuracy was higher for spectral CT than for conventional CT in colon tumors (77.8% vs 66.2%; χ² = 4.13, P = 0.042), whereas no statistically significant difference was observed in rectal tumors (61.8% vs 55.4%; χ² = 0.36, P = 0.551). In stage-grouped analyses, spectral CT showed higher accuracy than conventional CT in both early-stage disease (81.8% vs 71.4%; χ² = 1.82, P = 0.177) and advanced-stage disease (69.8% vs 58.0%; χ² = 3.19, P = 0.074), although these subgroup-specific comparisons did not reach statistical significance. Interaction testing did not provide evidence that the relative performance difference between modalities varied by tumor location (P for interaction = 0.548) or by stage grouping (P for interaction = 0.890) (**Table 6**).

**Table 6:** Subgroup analyses of overall TNM staging accuracy.

Subgroup	Spectral CT correct/total (%)	Conventional CT correct/total (%)	Test statistic	P value
Tumor location: colon (right + left)	91/117 (77.8)	88/133 (66.2)	χ² = 4.13	0.042
Tumor location: rectum	21/34 (61.8)	31/56 (55.4)	χ² = 0.36	0.551
Stage grouping: early (I–II)	45/55 (81.8)	50/70 (71.4)	χ² = 1.82	0.177
Stage grouping: advanced (III–IV)	67/96 (69.8)	69/119 (58.0)	χ² = 3.19	0.074

P for interaction (modality × location) = 0.548.

P for interaction (modality × stage grouping) = 0.890

CT, computed tomography; TNM, tumor–node–metastasis.

### 3.8 Sensitivity analyses

In the cohort restricted to patients with postoperative pathological TNM staging as the sole reference standard, overall TNM accuracy remained higher for spectral CT than for conventional CT (75.8% vs 63.1%; χ² = 4.81, P = 0.028). A similar pattern was observed when limiting the analysis to examinations performed within a strict pre-treatment interval of 14 days, with higher accuracy for spectral CT (76.7% vs 62.7%; χ² = 5.46, P = 0.019). Across both sensitivity analyses, the direction and magnitude of the between-modality difference were consistent with the primary analysis, supporting the stability of the main conclusion (**Table 7**).

**Table 7:** Sensitivity analyses for overall TNM staging accuracy.

Sensitivity analysis set	Spectral CT correct/total (%)	Conventional CT correct/total (%)	Test statistic	P value	OR (95% CI)
Surgical-pathology reference standard only	100/132 (75.8)	101/160 (63.1)	χ² = 4.81	0.028	1.83 (1.09–3.04)
Strict pre-treatment imaging interval (≤14 days)	92/120 (76.7)	94/150 (62.7)	χ² = 5.46	0.019	1.96 (1.14–3.35)

CI, confidence interval; CT, computed tomography; OR, odds ratio; TNM, tumor–node–metastasis.

## 4 Discussion

This retrospective comparison evaluated the staging performance of spectral CT versus conventional CT for CRC across overall TNM stage and individual T, N, and M components, using a reference standard primarily based on postoperative pathological TNM with predefined sensitivity analyses. In the main analysis, spectral CT yielded higher overall TNM accuracy than conventional CT and showed higher concordance with the reference standard for T and N staging, whereas M-staging performance was similar between modalities. Interobserver agreement was generally acceptable to good across staging components, with higher agreement for overall TNM and T staging than for N and M staging, and reproducibility of a continuous spectral parameter (iodine concentration) was supported by a high intraclass correlation coefficient.

The observed improvement in overall TNM accuracy with spectral CT was primarily driven by higher T- and N-staging performance. Accurate preoperative T classification is clinically relevant because it affects the selection and sequencing of treatment, including candidacy for neoadjuvant approaches in rectal cancer and surgical planning in colon cancer. Spectral CT outperformed conventional CT for T staging both in accuracy and in weighted agreement. These results align with an emerging body of work indicating that low-keV VMIs can increase lesion conspicuity and improve depiction of tumor margins and peritumoral changes, which are key elements in CT-based T staging (17, 18). For N staging, spectral CT also showed higher accuracy and higher κ than conventional CT. The binary analysis of nodal metastasis detection suggested broadly similar sensitivity between modalities, with higher specificity for spectral CT. This pattern is consistent with the concept that spectral-derived contrast enhancement quantification (e.g., iodine maps or iodine concentration) may assist in reducing false positives among borderline nodes, a recognized limitation of morphology-based CT nodal assessment. Radiomics approaches using dual-energy CT iodine maps have been reported for lymph node metastasis prediction in rectal cancer, supporting the rationale that iodine-related features carry incremental information beyond node size alone (19, 20).

By contrast, M-staging accuracy and agreement were comparable between groups. In routine CRC staging, contrast-enhanced CT of the thorax, abdomen, and pelvis remains a standard first-line approach for distant staging in many practice pathways, with MRI and FDG PET/CT often serving problem-solving or organ-specific roles (4). In this context, a ceiling effect is plausible: when conventional multidetector CT already provides high performance for many metastatic patterns (particularly when lesions are above typical detection thresholds and protocols are standardized), spectral CT may yield smaller incremental gains in overall M classification. Although overall M-stage accuracy was similar between the two modalities, the binary analysis suggested a different diagnostic balance, with spectral CT showing higher sensitivity but slightly lower specificity for M1 detection. This pattern may reflect improved conspicuity of subtle metastatic lesions on low-keV images, which could reduce under detection of distant disease, but may also increase the likelihood of false-positive interpretation for equivocal lesions. Clinically, higher sensitivity may be valuable in initial staging because missed metastases can substantially alter treatment planning, whereas the modest reduction in specificity indicates that some suspicious findings may still require confirmation with additional imaging or follow-up (21, 22). Low-keV VMI has been reported to improve iodine contrast in the liver, which is relevant to detecting liver metastases and characterizing small hepatic lesions, but downstream clinical impact depends on lesion prevalence, lesion size distribution, and verification pathways (4, 23).

Recent studies focusing on dual-layer or dual-energy/spectral techniques in CRC provide context for the present results. In a 2025 investigation of dual-layer spectral CT-derived VMIs combined with multiplanar reformation and evaluation of peritumoral features, the authors reported improved diagnostic performance for CRC T staging using low-keV VMIs, supporting the feasibility of spectral-based strategies for local staging refinement (24). A 2023 report in Radiologia Medica also discussed that virtual monoenergetic dual-layer dual-energy CT may improve CT diagnosis in CRC, and subsequent work has evaluated deep learning image reconstruction to facilitate reduced-dose low-keV imaging, which is relevant when considering the balance between image quality and radiation exposure in spectral protocols (25).

For nodal staging, multiple publications have explored quantitative or radiomics-based features derived from iodine maps to predict lymph node metastasis in rectal cancer, reflecting continued methodological development in an area where CT alone has historically shown constrained performance due to overlap between benign reactive nodes and metastatic nodes (19, 26). The moderate κ values for N staging in the present study, despite improvement with spectral CT, are consistent with the ongoing challenge of nodal assessment using imaging criteria. For rectal cancer, contemporary guidance emphasizes that high-resolution MRI provides diagnostic and prognostic information for local staging, including assessment of the mesorectal fascia and extramural vascular invasion; CT is generally not considered the primary modality for local rectal staging, although it remains important for distant staging (27–29). This framework helps interpret the subgroup analysis: the smaller absolute advantage of spectral CT in rectal tumors relative to colon tumors may reflect the inherent limitations of CT-based pelvic local staging compared with MRI-based approaches, particularly for fine discrimination between intermediate T categories and for mesorectal compartment assessment (30, 31).

The location-stratified analysis suggested that the improvement in overall TNM accuracy with spectral CT was more apparent for colon tumors than for rectal tumors, although interaction testing did not support statistically significant effect modification. This pattern is clinically plausible because CT-based evaluation of the colon wall, pericolic fat invasion, and adjacent organ involvement may benefit more from enhanced contrast resolution and improved conspicuity of peritumoral fat stranding on low-keV or iodine images than the complex pelvic anatomy encountered in rectal cancer, where MRI provides a more direct assessment of local extent and surgical planes (32). Sensitivity analyses restricted to cases with postoperative pathological staging and restricted to a strict pre-treatment imaging interval yielded results consistent in direction and magnitude with the primary analysis, supporting internal robustness. To further address the concern that the non-random assignment of imaging modality may have introduced residual confounding by indication, we performed multivariable logistic regression analyses using correct overall TNM staging as the dependent variable. After adjustment for age, sex, tumor size, tumor location, histologic differentiation, reference-standard stage grouping, and treatment intent, spectral CT remained independently associated with higher odds of correct overall TNM staging compared with conventional CT. This association remained materially unchanged after additional adjustment for reference standard type, supporting the robustness of the primary finding. These adjusted analyses suggest that the observed advantage of spectral CT was not fully explained by measurable differences in case mix alone. The sensitivity analyses specifically address two common sources of bias in retrospective imaging studies: heterogeneity in reference standards (particularly for non-resected patients) and temporal discordance between imaging and definitive treatment that can introduce stage migration. The multivariable logistic regression analysis further addressed the possibility of residual confounding related to non-random modality assignment. Interobserver agreement was high for ordinal outcomes (overall TNM and T stage) and lower for N and M stage, which is consistent with the relative subjectivity of nodal and small-lesion metastatic assessment (33). The high ICC for iodine concentration suggests that the continuous spectral parameter used in this study can be measured reproducibly under the applied protocol, which is relevant for standardization and potential incorporation into staging workflows or future quantitative models. Recent literature has increasingly treated iodine-derived parameters as reproducible surrogates of enhancement and tumor perfusion-related characteristics, supporting their use as quantitative inputs for prediction models, although the relationship between such parameters and histopathologic correlates remains context-dependent (34–36).

Several limitations should be considered when interpreting these findings. First, this was a retrospective study, and residual confounding by indication cannot be fully excluded despite broadly comparable baseline characteristics and consistent adjusted analyses; referral patterns, scanner availability, and clinical indications may still have influenced group composition. Second, complete blinding to imaging modality was not feasible. Although anonymization, withholding of non-essential clinical information, independent reading by two radiologists, and senior adjudication were used to reduce expectation bias, some effect of modality awareness, particularly for more subjective assessments such as T staging, cannot be excluded. Because reader-level uncertainty about modality recognition was not prospectively recorded, a reliable retrospective subgroup analysis was not possible. Third, the study compared two CT approaches rather than the full multimodality staging pathway. This is particularly relevant in rectal cancer, where pelvic MRI is generally preferred for local staging; in our cohort, MRI was available only in a subset of patients, and the relatively small rectal subgroup may have limited power to detect between-modality differences. Fourth, nodal assessment remained primarily morphology-based and only moderately concordant even with spectral CT. Quantitative spectral parameters and iodine maps were not systematically incorporated into node-level staging, and their specific contribution to nodal adjudication or interobserver agreement in equivocal cases was not prospectively assessed. Finally, although sensitivity analyses supported internal robustness, some verification bias may persist in non-surgical patients who required composite reference standards. Future studies should use prospective, preferably multicenter designs with standardized reading workflows, systematic node-level spectral quantification, prospective recording of reader confidence and modality-related uncertainty, and clearer integration of pelvic MRI in rectal cancer staging.

## 5 Conclusion

In this retrospective cohort, spectral CT demonstrated higher overall TNM staging accuracy than conventional CT, driven by improvements in T and N staging, while M staging performance was similar between modalities. Agreement with the reference standard and interobserver reliability were high for ordinal outcomes, and a continuous spectral enhancement parameter showed high reproducibility. These findings support further prospective evaluation of spectral CT within standardized CRC staging pathways to address persistent limitations in nodal assessment.

## Data Availability

The raw data supporting the conclusions of this article will be made available by the authors, without undue reservation.
