# Supplementary material for: Comparative accuracy of spectral computed tomography and conventional computed tomography in colorectal cancer staging
Source: Front Oncol. 2026 Jun 11;16:1802950. doi: 10.3389/fonc.2026.1802950 (PMC13293830; doi:10.3389/fonc.2026.1802950)
Supplement: Supplementary file 1 [file Table1.docx]

Supplementary Table S1. Multivariable logistic regression analyses for correct overall TNM staging

| Variable | Model 1 aOR (95% CI) | P value | Model 2 aOR (95% CI) | P value |
| --- | --- | --- | --- | --- |
| Imaging modality: spectral CT vs conventional CT | 1.91 (1.14–3.19) | 0.014 | 1.87 (1.10–3.17) | 0.019 |
| Age, per 1-year increase | 0.99 (0.97–1.02) | 0.564 | 0.99 (0.97–1.02) | 0.587 |
| Male sex | 1.07 (0.65–1.76) | 0.789 | 1.05 (0.63–1.75) | 0.847 |
| Tumor size, per 1-cm increase | 0.94 (0.83–1.07) | 0.344 | 0.95 (0.83–1.08) | 0.401 |
| Tumor location: rectum vs colon | 0.71 (0.40–1.24) | 0.227 | 0.73 (0.41–1.29) | 0.277 |
| Histologic differentiation: poor vs well/moderate | 0.82 (0.47–1.42) | 0.48 | 0.84 (0.48–1.48) | 0.551 |
| Stage grouping: III–IV vs I–II | 0.58 (0.34–0.98) | 0.042 | 0.60 (0.35–1.03) | 0.063 |
| Treatment intent: neoadjuvant/non-surgical vs upfront surgery | 0.63 (0.35–1.13) | 0.121 | 0.79 (0.36–1.71) | 0.547 |
| Reference standard type: composite vs surgical pathology only | — | — | 0.76 (0.40–1.44) | 0.398 |

aOR, adjusted odds ratio; CI, confidence interval; CT, computed tomography; TNM, tumor–node–metastasis. The dependent variable was correct overall TNM staging (1 = exact agreement between imaging-based overall TNM stage and the reference standard; 0 = disagreement). Model 1 was adjusted for age, sex, tumor size, tumor location, histologic differentiation, stage grouping, and treatment intent. Model 2 additionally included reference standard type.
